# Physical activity and sedentariness levels in patients with post-exertional malaise resulting from post-COVID-19 syndrome

**DOI:** 10.1177/10519815251329231

**Published:** 2025-04-28

**Authors:** Kamel-Eddine Elkebir, Jo-Anne Gilbert, Thiffya Arabi Kugathasan, Camille Cazeneuve, Florian Chouchou, Marie-Eve Mathieu

**Affiliations:** 1 School of Kinesiology and Physical Activity Sciences, Université de Montréal, Montréal, Canada; 2 Diabète Athérothrombose Réunion Océan Indien (DéTROI), INSERM UMR 1188, Campus Santé de Terre Sainte, Université de La Réunion, Saint-Pierre, France; 3 IRISSE Laboratory (EA4075), UFR SHE, University of La Reunion, La Réunion, France; 4 Azrieli Research Center, Sainte-Justine University Hospital Center, Montréal, Canada

**Keywords:** sedentary behavior, infection, workers, sex, walk, bike

## Abstract

**Background:**

Post-exertional malaise (PEM) is a complex phenomenon characterized by extreme fatigue, reduced endurance, and muscular and joint pains. Physical activity (PA) has recognized health benefits, including reducing the risks of chronic diseases and mortality. During the pandemic, a general decline in PA was measured, but the profile of the various components of PA and sedentariness in patients with PEM resulting from post-COVID-19 syndrome (PCS-19) remains scarce. It is relevant to observe the impact of these discomforts on PQ after their occurrence.

**Objective:**

This study examines the detailed PA and sedentary profile of individuals affected by PEM associated with PCS-19.

**Methods:**

An online questionnaire disseminated via social media platform evaluated PA and sedentariness before and after COVID-19 diagnostic.

**Results:**

Individuals with PEM (n = 154) became more sedentary and inactive post-COVID-19. Specifically, PA at work decreased in women and those whose last infection occurred over a year ago. Walk decreased for women but increased for men. Bike journeys generally decreased after COVID-19. The severity of PEM, the pace of recovery, and fear of malaise influenced PA changes.

**Conclusions:**

The PCS-19 leads to increased sedentary behavior and a decline in PA, particularly at work, and is more pronounced among women and those more severely affected by PEM. These findings are critical for post-COVID PA resumption, including for workers who go back to work and who regain normal duties while being potentially deconditioned.

## Introduction

COVID-19 is an infection with the SARS-CoV-2 virus that ranges from an infection that is asymptomatic to a mild respiratory ailment, which can progress to severe forms in certain individuals, especially the elderly and those with pre-existing health issues.^1^ Common symptoms include nasal congestion, fatigue, fever, loss of smell and taste, and cough.^2^ Ghafuri, Lida et al. [2021] proposed a simple clinical classification of severity levels: mild, moderate, severe, and critical, based on clinical manifestations, test results, and organ involvement. Patients with mild forms typically recover without hospitalization, while moderate cases may require hospitalization and oxygen therapy. Severe and critical cases often necessitate intensive care, mechanical ventilation, and support for failing organs.^3^

The post-COVID-19 syndrome (PCS-19) encompasses persistent symptoms felt for weeks or even months after the initial COVID-19 infection. It is estimated that between 10% and 35% of patients showing symptoms of COVID-19 develop PCS-19, a rate that can reach up to 85% for hospitalized patients.^4,5^ The most frequently observed complications in people with the syndrome fall into three categories: persistent fatigue, respiratory and physical sequelae, and post-traumatic stress disorder and psychiatric symptoms.^6^ The underlying mechanisms of these complications are multifactorial and may involve biological, immunological, neurological, and environmental factors.^6,7^

Post-exertional malaise (PEM) is a complex phenomenon commonly found in patients with myalgic encephalomyelitis/chronic fatigue syndrome but also in PCS-19.^8,9^ It is characterized by extreme fatigue, reduced endurance, and muscular and joint pains.^10^ Patients with myalgic encephalomyelitis/chronic fatigue syndrome and PCS-19 show similar symptoms, prolonged symptom duration, and reduced daily activity.^9^ The mechanisms behind PEM are still poorly understood but may involve central, peripheral, and psychological factors.^11^ PEM can occur immediately after activity or be delayed, and its duration varies among individuals. Preventive measures such as rest, appropriate activity management, and good sleep hygiene are generally beneficial for managing fatigue symptoms related to PEM.^12^

Physical activity (PA) has recognized health benefits, including reducing the risks of chronic diseases and mortality.^13–15^ However, during the COVID-19 pandemic, opportunities for PA were reduced, leading to a decrease in PA and an increase in sedentary behavior.^16,17^ Studies show that active individuals are less likely to contract COVID-19 and suffer from severe complications, potentially due to its impact on mental health and the very symptoms of PCS-19.^18^ Thus, during the recent pandemic, it was recommended to regularly engage in moderate-intensity aerobic activity, like brisk walking, while adhering to preventive measures and health guidelines.^19,20^ In fact, PA is beneficial for immunological health, to manage and mitigate physical syndromes, is an effective treatment for pulmonary complications, improves cardiovascular health, stimulates brain plasticity, and increases psychological well-being.^21^ However, an alteration in active lifestyles has been observed in many different populations during the pandemic period. For the elderly, a meta-analysis of 25 studies, including 14 cross-sectional and 11 cohort studies, showed that they were particularly vulnerable during the restrictions induced by the pandemic. Their PA decreased markedly, leading to a deterioration in fitness and accentuating sedentary lifestyles, which amplified the risks of frailty.^22^ For children, an in-depth review of 1672 studies, 84 of which met the inclusion criteria, highlighted the profoundly negative effects of COVID-19 restrictions on children's PA. PA decreased significantly, between 10.8 and 91 min per day, during the pandemic. Where there was an increase, it generally concerned unstructured outdoor activities.^23^ Such changes are not without consequences as shown in Spain in a longitudinal study that spanned the lockdown period confirmed reduction in PA but also showed a deterioration in several health metrics such as sleep quality and well-being among physically active adults.^24^

However, the profile of the various components of PA (work, leisure, and active transportation) and sedentariness in patients with PEM resulting from PCS-19 remains scarce. Thus, this study aims to analyze the PA and sedentary profile of patients with PEM. We hope to shed light on the path forward for optimal management of these patients and better understand the repercussions of PCS-19 on the patients’ ability to lead an active life.

## Methods

The cross-sectional study was conducted using a questionnaire to assess in detail PA and sedentariness in individuals suffering from PCS-19 and, more specifically, PEM. Eligible participants were adults aged 18 and over, with symptoms of PCS-19, no history of chronic fatigue syndrome before COVID-19 and having contracted COVID-19 for the first time at least three months ago. The online questionnaire was available in both French and English via “LimeSurvey GmbH” between July 21 and September 21, 2022. Recruitment was done via Facebook and targeted an international audience. An information sheet explaining the objectives of the study was published along with the links leading to the questionnaires. The ethics approval was obtained from the University of Montreal's Health Research Ethics Board (2021-374). Consent and eligibility of the participants were obtained online. A sample of 128 participants was calculated based on a pilot study to detect the risk of PEM among individuals with PCS-19 with a power of 80%.

The questionnaire consisted of 5 parts, addressing the period before and after contracting COVID-19. PEM was assessed using the DePaul Symptom Questionnaire - Post-Exertional Malaise Short Form.^25^ In this questionnaire, the patient is at risk if at least one of the five questions has a score of 2 to 4 for frequency combined with a score of 2 to 4 for severity of the same item. The questionnaire demonstrated a good ability to correctly categorize ME/CFS patients based on their PEM symptoms. These items showed good internal and test-retest reliability. PA levels and sedentariness were measured using the ONAPS-PAQ questionnaire, which includes questions about moderate-to-vigorous PA (MVPA) at work, active transport, and leisure/home activities.^26^ For sedentariness questions, a subject was considered mildly sedentary if the total duration of sedentary activity did not exceed three hours per day, moderately sedentary when they had three to seven hours of sedentary activity per day, and more than seven hours were considered high levels of sedentariness.^26^ Sociodemographic questions were asked to characterize the participants.

Questionnaire data were analyzed by excluding participants 1) who interrupted the questionnaire before the end of the last question, 2) who refused consent, 3) showing a risk of PEM before COVID-19, or 4) reporting unusual amount of PA (i.e., > 7000 min of PA per week). Descriptive statistics with mean values (standard deviation) were used to describe the sociodemographic characteristics of the study population. General linear tests with repeated measures were applied to analyze the relationship between lifestyle, risk of PEM, and sociodemographic characteristics. Independent t-tests were conducted to compare groups based on sociodemographic data and PEM parameters before and after COVID-19. Paired t-tests were performed to assess the differences within the same group before and after COVID-19. Finally, an eta squared analysis was carried out to determine the effect size, using Cohen's coefficient, to interpret the influence of the independent variable on the dependent variable. Analyses were performed using SPSS 28.0.1.0 (SPSS Inc., Chicago, IL, USA).

## Results

One hundred sixty-seven individuals filled the questionnaire, of which 154 (92.22%) were considered at risk for PEM and included in the current study. The average age of participants at risk for PEM was 45.8 (standard deviation: 10.5) years. Participants at risk for PEM were predominantly female (86.2%). The majority of those at risk for PEM had contracted COVID-19 once (63.6%), compared to those who had it 2 to 3 times (31.8%) and those who had it more than 3 times (4.5%). An average of 440 (275) days elapsed since they contracted COVID-19.

Significant interactions were observed between sex at birth and the period (pre/post COVID-19) for MVPA at work, total MVPA, active transportation (walk and bike) (Table 1). Post-hoc analysis revealed that women had higher levels of MVPA at work before COVID-19 than men (p = 0.006) and that the reduction in MVPA at work following COVID-19 was significant in women (p < 0.001). For total PA, MVPA (p < 0.001) was reduced in women in the post-COVID period. Regarding active transportation, women significantly reduced their use of walk (p < 0.0001) and bike (p = 0.001) in the post-COVID period. Similarly, before COVID-19, men cycled more on average than women, but after COVID-19, both groups cycled much less on average (p < 0.001). In terms of the time effect, a statistically significant decrease was observed for leisure-time MVPA (p < 0.001) while for sedentariness, statistically significant increases were observed (p = 0.036) following COVID-19. About the group effect, sedentariness showed a statistically significant difference (p = 0.006), with men being more sedentary than women, both before and after COVID-19.

**Table 1.** Duration of physical activity before and after COVID-19, by sex at birth.

Type of activity	Before COVID-19		After COVID-19		Interaction		Time		Group	
	Women n = 131	Men n = 21	Women n = 131	Men n = 21	p	Eta-square	P	Eta-square	p	Eta-square
MVPA work, min/week	1015 (1189)	418 (797) ^#^	298 (728) *	307 (953)	**0**.**021**	0.032				
MVPA leisure, min/week	465 (524)	486 (401)	75 (286)	45 (131)	0.696	0.001	**< 0.001**	0.214	0.953	0.000
Active transport-Walk, min/week	419 (616)	392 (502)	133 (416) *	865 (2751)	**0**.**003**	0.050				
Active transport-Bike, min/week	37 (125)	346 (1305)	1 (10) *	17 (79)	**0**.**012**	0.041				
MVPA total, min/week	1937 (1600)	1643 (1699)	508 (1098) *	1235 (2979)	**0**.**023**	0.034				
Sedentariness, min/day	465 (206)	611 (574)	504 (355)	766 (752)	0.204	0.011	**0.036**	0.029	**0**.**006**	0.050

MVPA: Moderate-to-vigorous physical activity. ^#^: significantly different from the other group at the same timepoint (post-hoc analysis). *: significantly different from the other timepoint for the same group (post-hoc analysis). Significance threshold set at p < 0.05.

Analyses revealed significant interactions for MVPA at work (p = 0.012) (Table 2). In both groups, the results showed significant decreases in MVPA at work (1 year and under: p < 0.001 and over 1 year p < 0.001). About the time effect, statistically significant results were observed for leisure MVPA (p < 0.001) and total MVPA (p < 0.001) where a decrease was monitored over time.

**Table 2.** Duration of physical activity before and after COVID-19, as a function of time elapsed since COVID-19.

	Before COVID-19		After COVID-19		Interaction		Time		Group	
Type of activity	≤1 years n = 113	>1 year n = 40	≤1 years n = 113	>1 years n = 40	p	Eta-square	p	Eta-square	p	Eta-square
MVPA work, min/week	863 (1120)	1126 (1260)	363 (824) *	112 (489) ^#,*^	**0**.**012**	0.041				
MVPA leisure, min/week	449 (492)	523 (555)	72 (298)	64 (168)	0.422	0.004	**< 0.001**	0.309	0.544	0.002
Active transport-Walk, min/week	402 (611)	453 (575)	186 (506)	364 (1988)	0.556	0.002	0.159	0.013	0.349	0.006
Active transport-Bike, min/week	95 (578)	34 (82)	4 (35)	1 (7)	0.528	0.003	0.179	0.012	0.494	0.003
MVPA total, min/week	1809 (1582)	2137 (1712)	625 (1255)	543 (2075)	0.248	0.009	**< 0.001**	0.290	0.588	0.002
Sedentariness, min/day	484 (313)	487 (200)	558 (461)	509 (376)	0.474	0.003	0.191	0.011	0.692	0.001

MVPA: Moderate-to-vigorous physical activity. ^#^: significantly different from the other group at the same timepoint (post-hoc analysis). *: significantly different from the other timepoint for the same group (post-hoc analysis). Significance threshold set at p < 0.05.

Pre- and post-COVID-19 PA as a function of PEM variables highlighting statistically significant interactions in several areas. For the risk of PEM, interactions for MVPA at work (p = 0.003), as well as for sedentariness (p = 0.014) were obtained (Table 3a). Post-hoc analysis reveals a marked distinction in terms of MVPA at work post-COVID-19 between the group at risk on fewer than 5 questions of the PEM questionnaire compared with those at risk on all 5 questions (p = 0.042). The results also highlight that the group at risk on all 5 questions experienced a significant decrease in MVPA at work (p < 0.001) when comparing the pre- and post-COVID-19 periods. In terms of sedentariness, analysis shows that it is in the group of people at risk on fewer than 5 questions of the PEM questionnaire that there is a significant increase (p = 0.027) when comparing pre- and post-COVID-19 periods. In terms of the time effect, statistically significant results were observed for leisure MVPA (p < 0.001) and total MVPA (p < 0.001), all pointing to a reduction in active lifestyle after COVID-19.

**Table 3.** Duration of physical activity before and after COVID-19, according to questions answered about post-exertion discomfort.

Type of activity	Before COVID-19		After COVID-19		Interaction		Time		Group	
*a) Risk PEM (5Q filled or less < 5Q filled)*										
	< 5Q n = 27	5Q n = 127	< 5Q n = 27	5Q n = 127	p	Eta-square	p	Eta-square	p	Eta-square
MVPA work, min/week	727 (1037)	976 (1179)	660 (1092)	218 (642)*	**0**.**003**	0.014				
MVPA leisure, min/week	455 (502)	469 (510)	88 (154)	66 (288)	0.756	0.001	**< 0.001**	0.222	0.948	0.000
Active transport- Walk, min/week	392 (478)	420 (623)	148 (321)	248 (1198)	0.766	0.001	0.096	0.018	0.645	0.001
Active transport- Bike, min/week	19 (47)	92 (547)	4 (19)	3 (32)	0.488	0.003	0.329	0.006	0.495	0.003
MVPA total, min/week	1593 (1410)	1957 (1651)	900 (1208)	537 (1553)	0.073	0.021	**< 0.001**	0.153	0.998	0.000
Sedentariness, min/day	427 (205)	496 (300)	654 (507) *	520 (421)	**0**.**014**	0.039				
*b) Recovery time*										
	< 24h n = 47	≥ 24 n = 107	< 24h n = 47	≥ 24 n = 107	p	Eta-square	p	Eta-square	p	Eta-square
MVPA work, min/week	683 (887)	1042 (1244)^#^	332 (704) *	279 (779) *	**0**.**036**	0.027				
MVPA leisure, min/week	550 (610)	430 (452)	70 (166)	70 (304)	0.214	0.010	**< 0.001**	0.334	0.249	0.009
Active transport- Walk, min/week	401 (512)	422 (635)	407 (1842)	155 (492)	0.181	0.012	0.202	0.011	0.321	0.006
Active transport- Bike, min/week	52 (186)	91 (584)	3 (15)	4 (33)	0.663	0.001	0.121	0.016	0.646	0.001
MVPA total, min/week	1686 (1329)	1985 (1720)	811 (1967)	508 (1243)	0.073	0.021	**< 0.001**	0.247	0.992	0.000
Sedentariness, min/day	475 (214)	488 (314)	557 (422)	537 (448)	0.633	0.002	0.059	0.023	0.952	0.000
*c) Fear of post-exertion discomfort*										
	No Afraid n = 10	Afraid n = 144	No Afraid n = 10	Afraid n = 144	p	Eta-square	p	Eta-square	p	Eta-square
MVPA work, min/week	181 (473)	984 (1172)^#^	349 (909)	292 (747) *	**0**.**018**	0.024				
MVPA leisure, min/week	282 (187)	479 (520)	22 (37)	73 (278)	0.418	0.004	**< 0.001**	0.082	0.203	0.011
Active transport- Walk, min/week	608 (899)	402 (574)	67 (137)	243 (1132)	0.318	0.007	0.069	0.022	0.946	0.000
Active transport- Bike, min/week	160 (386)	73 (505)	36 (114)	1 (9)	0.750	0.001	0.231	0.009	0.459	0.004
MVPA total, min/week	1231 (1168)	1939 (1632)	474 (904)	609 (1535)	0.363	0.005	**0**.**001**	0.068	0.295	0.007
Sedentariness, min/day	526 (225)	481 (290)	724 (693)	531 (416)	0.251	0.009	0.056	0.024	0.250	0.009

MVPA: Moderate-to-vigorous physical activity. ^#^: significantly different from the other group at the same timepoint (post-hoc analysis). *: significantly different from the other timepoint for the same group (post-hoc analysis). Significance threshold set at p < 0.05.

For the recovery time, the interaction for MVPA at work was significant (p = 0.036) (Table 3b): a difference between the two groups pre-COVID-19 was observed (p = 0.045), with a higher level of MVPA at work for those taking longer than 24 h to recover from PEM. Post-COVID-19, both groups recorded a decrease in MVPA at work (p < 0.001 in both cases). In terms of the time effect, reductions were observed for leisure MVPA (p < 0.001) and MVPA total (p < 0.001).

Finally, for the fear of post-exertion discomfort, interactions for MVPA at work was measured (p = 0.018) (Table 3c). There was a greater difference in terms of MVPA at work prior to COVID-19 between the fearful and non-fearful groups (p < 0.001), with individuals being afraid of having higher MPVA at work. About the time effect, statistically significant results were observed for leisure MVPA (p < 0.001) and total MVPA (p = 0.001). These results indicate a general reduction in PA after COVID-19.

## Discussion

The recent pandemic left many individuals with limitations and PEM is being one of them. Previous studies showed that PEM post-Covid-19 led to lower overall PA levels and higher sedentariness than PEM linked to chronic fatigue syndrome.^27,28^ The current study is the first to document changes in various components of PA and sedentariness in patients with PEM and to investigated associated characteristics of participants. Compared to before infection, individuals with PEM changed PA at work, in leisure time, in transport and sedentary time after COVID-19. However, some distinctions emerged with women being more affected for work and total MVPA while men showed an increase in walk for transportation but a reduction in cycling for transportation. While the duration since the infection is not associated with changes in PA levels, with some changes persisting even for those infected for more than one year, factors such as the gravity of PEM, recovery time from PEM and fear of post-exertion discomfort are important to consider.

### Work

Women who answered to the questionnaire engaged in higher levels of MVPA at work before the pandemic than men. This finding is not expected given that men usually exhibit higher physical involvement at work than women.^29^ It could be due to the fact that women with more physical work experienced to a larger extent PEM. This aligns well with the observation by Sudre et al. [2021] who reveals that women are more susceptible than men to present persistent symptoms.^30^ The onset of the PCS-19 led to a decrease of about two-third in work related MVPA in women. This finding aligns well with the fact that reduction in PA is noticed more in active individuals.^31^ Work-related MVPA showed a decline post-COVID-19 in those who had been infected more recently (1 year or less) and for a longer period (over one year). This decline is concerning as it suggests not only short-term but also long-term impacts on the physical functioning of individuals in their work environments. A dose-response relationship also emerged from our findings. In fact, the gravity of the PEM is also associated with the decline of MVPA at work as shown by a five-time reduction in activity levels when the participants had 5 positive scores on the post-exertion discomfort scale while it remains stable with less than 5 positive scores. The longer it takes to recover from a PEM was also associated with higher reduction in MVPA at work. Finally, individuals manifesting a fear of post-exertion discomfort were those with more active jobs at baseline. It is possible that individuals anticipate more PEM if their usual job is physically demanding.

### Leisure

Following COVID-19, a reduction in leisure MVPA was observed to a similar extent in all subgroups of interest: men and women, in individuals who were affected recently (<=1 year) and since a while (> 1 year), in those with low and high risk of PEM, in those with short or long PEM recovery time and in those with or without fear of post-exertion discomfort. While work MVPA was usually double in volume compared to leisure MVPA before COVID-19, leisure MVPA could be 15 times lower than what was observed for work MVPA post-COVID-19 in participants with PEM. This larger difference could be due to the fact that work PA is not fully under the individual control while leisure PA is. It can also be hypothesized that participants with PEM which returned to work thus haven’t prioritized yet leisure PA. It is important to highlight that intensity is one important factor to consider when a professional take in charge PCS19 patients and that PA programs needs to the adapted and progressive.^32^ Protocols such as the one from the World Health Organization might be interesting to follow.^19^ The importance of leisure PA is also high considering that remote work increased since the pandemic^33^ and that workers might need to rely more on leisure PA to expend energy previously devoted to active transportation to work and on-site work.

### Active transport

The pandemic period was associated with a period of change in commuting mode in the general population affecting about 50% of participants.^34^ In our study conducted with patients with PEM, active transportation, which includes walking and biking, also underwent changes following COVID-19. There was a decrease in walking for active transportation among women, whereas men showed an increase. Lower active commute in women during pandemic is in accordance with previous studies.^34^ Bike use generally decreased, suggesting a shift from cycling to walking for men. It is aligned with findings that bike-sharing systems were less used during the pandemic.^35^ Reintroduction of active transportation should be done along with consideration of work and leisure PA to avoid overtraining. To do so, the inclusion of rest periods, a strategy to consider to avoid exacerbation of PEM symptoms,^9^ could be of interest.

### Sedentariness

COVID-19 pandemic induced a general increase in sedentariness due to confinement and restrictions in mobility.^36,37^ In our sample, sedentariness increased following COVID-19, but to a factor below two. This behavior was more pronounced in men than in women both before and after COVID-19. The increased sedentariness is concerning as it is associated with negative physical and mental health outcomes^31,38^ and may compound the challenges faced by individuals with PEM in recovering from the effects of COVID-19.^31^ While reduction in sedentariness is desired and encouraged, notably reduced screen time,^31^ it can yield to a shift towards light and/or more intense PA that need to be considered from a global point of view, potentially more for PEM patients to avoid negative outcomes associated to enhanced physical exertion.

### Strengths and limitations

The present study explores the health consequences of COVID-19, including PEM, using an online questionnaire to collect data. This method facilitated access to a wide audience and reduced the risk of contamination. The study considered variations among participants according to sex at birth, age and socio-demographic status, offering an in-depth perspective on post-COVID-19 PA behaviors. However, recruitment challenges for some participants, the possible inaccuracy of self-reports and the use of non-validated questionnaires in English or French are limitations. Additionally, the modest number of male participants (n = 21) compared to female participants (n = 131) may limit the generalizability of the results to a broader population, as it may not fully capture the variations in post-COVID-19 health consequences across different genders. This demographic imbalance underscores the need for further research to ensure comprehensive understanding across all sex demographics. Also, the study was unable to compare an PEM group with a non-PEM group because the sample size of the latter was n = 13. At this stage, several avenues of research remain to be explored. It would be beneficial to conduct longitudinal studies to track variations in PA levels and PEM symptoms over the long term, including follow-ups. In addition, research could focus on the creation and evaluation of personalized interventions, such as supervised home exercise or tele-rehabilitation, to meet the specific needs of PEM patients and improve their quality of life.

## Conclusion

The study aimed to define the PA profile and sociodemographic data of people with PCS-19 at risk of PEM. Results showed a decrease in MVPA levels at work for people with PCS-19 with PEM, especially for those most severely affected by PEM. In addition, women reduced their levels of MVPA to a larger extent than men while the duration since the contamination did not emerge as a factor to consider beyond the fact that MVPA was lower after contamination. In addition, this research highlights the importance of occupational health promotion after COVID-19, emphasizing the need for tailored programs to minimize the risk of PEM, provide a safe transition towards an active lifestyle at work and in daily life. Resources, including professional guidance in the workplace, might be needed for workers with PEM to avoid further deconditioning and exacerbation of PEM.
